# Risk factors for hyperactive *delirium* among icu adult patients in southern brazil: a prospective cohort study

**DOI:** 10.1186/2197-425X-3-S1-A331

**Published:** 2015-10-01

**Authors:** RPd Oliveira, R Rosa, A Ascoli, W Rutzen, L Madeira, P Balzano, P Morandi, V Souto, F Vargas, L Lago, C Dietrich, J Rezende, F Ghizzoni, MC Souza, C Guterres, M Falavigna, K Lima, C Robinson, R Ribeiro, J Maccari, C Teixeira

**Affiliations:** Moinhos de Vento Hospital, Porto Alegre, Brazil

## Introduction

*Delirium* is associated with worse outcomes among intensive care unit (ICU) patients*.* Data about risk factors for *delirium* in Latin American ICUs are scarce.

## Objectives

To determine risk factors associated with hyperactive delirium among ICU adult patients in Southern Brazil.

## Methods

A multicenter prospective cohort study was performed with all consecutive adult patients admitted to mixed medical-surgical ICUs in Southern Brazil between May 2014 and December 2014. The present study is part of BaSICS (Brazilian Study of Post Intensive Care Syndrome). Hyperactive *delirium* was diagnosed by the confusion assessment method for the intensive care unit (CAM-ICU). A stepwise logistic regression was conducted to determine factors associated with incidence of hyperactive *delirium* during ICU stay.

## Results

During the study period 124 patients were evaluated. The mean age and APACHE-II score were 64.5 years (SD 16.5) and 14.1 points (SD 5.3), respectively. The mean ICU length of stay was 8.8 days (SD 9.5). The overall incidence of hyperactive *delirium* was 25.8% (32 patients). Upon multivariate logistic regression analysis, parenteral sedation need during ICU stay (OR, 9.59; 95%CI, 3.42-26.9) and Charlson comorbidity index (OR, 1.33; 95%CI, 1.08-1.63) were risk factors for hyperactive *delirium*. On the other hand, admission in a single-bed ICU room (OR, 0.15; 95%CI, 0.04-0.50) was a protective factor against hyperactive delirium during ICU stay.

## Conclusions

Delirium in the intensive care unit (ICU) is exceedingly common, and risk factors for delirium among the critically ill are nearly ubiquitous. Nevertheless, maybe the action on the modifiable risk factors (eg, sedation management), and changes in architecture of the ICU could help to prevent the appearance of this syndrome.
